# Genome of tiny predator with big appetite

**DOI:** 10.1186/s12915-018-0610-8

**Published:** 2018-11-28

**Authors:** Geoffrey Ian McFadden

**Affiliations:** School of BioSciences, University of Melbourne, VIC 3010 Australia

## Abstract

The capture and enslavement of eukaryotic algae by unicellular predators to acquire photosynthesis was a major driving force in early eukaryotic diversification. A genome presented in *BMC Biology* provides a glimpse of how such a tiny predator might have preyed on red algae and detained them to create new lineages of photosynthetic organisms.

## Commentary

Few biologists will have heard of *Goniomonas*, though they could find this minute, colorless biflagellate in just about any stream or inlet they cared to look in. But why should they care? Well, *Goniomonas* is an important piece in the puzzle of how the colorful flags of photosynthesis came to be daubed across numerous different and unrelated branches of the tree of eukaryotes [1].

*Goniomonas* first came to my attention 25 years ago when friend and cryptomonad aficionado David Hill showed me a little ‘bug’ he had retrieved from the Yarra River in Melbourne. Once I had grasped what I was viewing, I knew we had to get its DNA and try to unravel how *Goniomonas* is related to the photosynthetic cryptomonads. Cryptomonads, you see, are the ‘smoking gun’ of a remarkable and massively influential evolutionary process known as *secondary endosymbiosis*.

Plastids—the subcellular structures in plants and algae where photosynthesis occurs—first arose by endosymbiosis of a cyanobacterium. The cyanobacterium was engulfed by a eukaryotic phagotroph, but instead of being digested for food as normally would happen to a phagotroph’s prey, it was detained as a solar cell to perform the metabolic magic of using light energy to smash water, release electrons, and string carbon atoms together to create the fuel of life—glucose. This initial endosymbiosis, referred to as the *primary endosymbiosis*, spawned the red algae, the glaucophytes, and the green algae and their eventual descendants, the plants. The plastids in these photosynthesizers have two bounding membranes, now clearly understood to derive from the pair of membranes that enclose your typical, Gram-negative cyanobacterium.

But primary endosymbiosis was merely the first chapter in the acquisition of photosynthesis by eukaryotes. Once the cyanobacterium was tamed, it was transplanted horizontally into several disjunct eukaryotic lineages by multiple secondary endosymbioses. These secondary endosymbioses—sometimes involving a red alga and sometimes involving a green alga, but curiously never a glaucophyte—resulted in the eukaryotic alga becoming the detainee in one or another phagotroph to create a metaorganism comprising a relic cyanobacterium inside a eukaryote inside yet another eukaryote. How do we know this with certainty? Cryptomonads. These little algae are a classic Darwinian transition form in this shell game, retaining all the shells of the two sequential engulfment processes, plus a few tell-tale bits and pieces of the swallowed ones.

The crucial piece in cryptomonads is the vestigial nucleus of the engulfed red alga, which we now refer to as the nucleomorph in recognition of Dennis Greenwood’s seminal observation that it had the morphology of a little nucleus sequestered between the shells (membranes) of the metaorganism (references in [2]). Nucleomorph genomes confirm that this little structure is indeed the relic nucleus of the red algal endosymbiont and prove unequivocally that secondary endosymbiosis did happen, and that cryptomonads are one result [3]. Nucleomorphs give credence to all the other putative secondary endosymbioses where the nucleomorph, and even some of the membrane shells, have apparently disappeared with reduction of the endosymbiont through increased integration into the metaorganisms.

## *Goniomonas* genomes and a window into the origins of photosynthesis

**Fig. 1.**
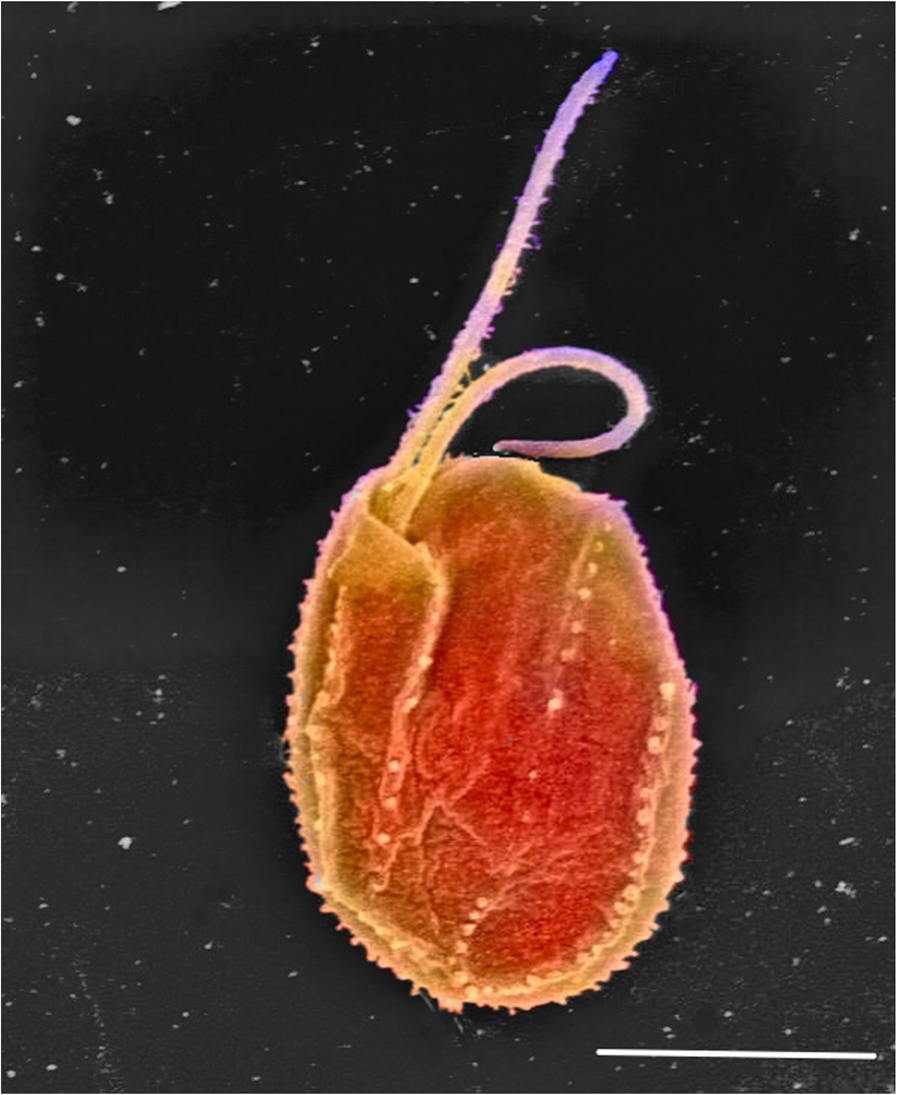
Scanning electron micrograph of the tiny predator *Goniomonas avonlea* (colourised). Typical cryptomonad features such as the flat, asymmetric shape, two anterior flagella emerging from a complex invagination, and the light armour plating are visible. Photo credit E. Kim. Bar = 5 μm

But back to *Goniomonas*. The German phycologist Georg Fresenius recognized *Goniomonas* as a cryptomonad 160 years ago on the basis of its asymmetric shape, presence of ejectisomes (enigmatic ejectile structures that burst like party streamers from the cell under duress), and the two unequal flagella emerging from a deep, complicated anterior invagination [4] (Fig. 1). Where *Goniomonas* differs to most cryptomonads—which are either blueish-greenish or reddish-brownish depending on which of the endosymbiont’s phycobilin pigments they retain—is it lacks any color, and apparently any plastid [5]. This raises the question of whether it had a plastid and carelessly lost it, or whether it is a representative of the cryptomonads before they entered into their complex secondary endosymbiotic relationship with a red alga? A first step would be to infer how *Goniomonas* relates to photosynthetic cryptomonads, and DNA sequences were the obvious approach.

To get DNA sequences from organisms one typically needs a goodly amount of them. *Goniomonas* is a predator, so when Hill and I wanted to grow it up in large numbers, we opted to provide it with loads of bacteria to engulf and digest—this was as simple as me popping a grain of wheat into several flasks of culture media to support bacterial growth, whilst Hill arduously plucked out individual *Goniomonas* cells with a micropipette and placed them in solitary confinement with only food for company. Hill and I got a clonal culture and a ribosomal RNA sequence from *Goniomonas truncata*, and could show it was sister to photosynthetic cryptomonads [6].

In their recent article in *BMC Biology*, Cenci et al. report the entire genome of a related species, *Goniomonas avonlea* [7], which hails from the setting of Lucy Montgomery’s novel *Anne of Green Gables.* The *G. avonlea* genome paints the first complete picture of how cryptomonads might have functioned before they crossed the tracks and made a pact with an alga to become autotrophic. It is a fascinating window into how things probably were with cryptomonads before they switched lifestyles.

As genomes go, the *G. avonlea* blueprint is still a bit of a roughie, remaining in ~ 32,000 unjoined pieces. That’s because the genome is huge for such a tiny organism, with a final size approaching 100 megabases; the effort to get it fully assembled and polished would be formidable. Gene count, at ~ 18,000 non-redundant proteins, is also impressive, and these genes are, on average, interrupted by about five introns each. But numbers aside, what does the genome tell us about how *G. avonlea* makes its living, now and in the past? Quite a bit as it happens. A concerted search turned up no convincing evidence for *G. avonlea* now having, or ever having had, a plastid. Thus, we can be pretty certain this type of cryptomonad is ancestrally heterotrophic. Metabolic pathways typically taken care of by the plastid in plants and algae—namely fatty acid synthesis, isoprenoid precursor synthesis, iron sulfur cluster generation, and heme synthesis—are apparently done in the cytosol of *G. avonlea* using canonical eukaryotic machinery not related to cyanobacteria. Thus, there are no traces of plastid-type metabolisms lurking in the genome, and *Goniomonas* appears to be a living representative of the pre-secondary-endosymbiosis cryptomonads.

## An ancestral phagotroph?

*G. avonlea* also seems well equipped gene-wise to digest its prey, having a panoply of lysozymes to chew through the cell walls of those bacteria unfortunate enough to end up going down the gullet of the predator and into its food vacuoles. For me though, the jewel-in-the-crown of the *G. avonlea* genome are glycan hydrolases belonging to the GH50 family, which cleave β-1,4 glycosidic bonds of agarose, a principal component of red algal cell walls. *G. avonlea* thus seems equipped to be algivorous, capable of digesting the walls of red algal prey cells. In today’s oceans and streams, most red algae are multicellular and too large to be preyed upon by a miniscule flagellate like *G. avonlea*, but unicellular red algae small enough for a *Goniomonas* cell to engulf are not uncommon and were perhaps more so in earlier times. Thus, if *G. avonlea* does indeed engulf and digest red algae, it is not too much of a stretch to imagine a scenario where the prey cell is detained and not digested—exactly the kind of event predicted to have occurred at the outset of a secondary endosymbiosis creating the photosynthetic cryptomonads [2, 3].

*Goniomonas* is thus a nice fit for the ancestral phagotroph that was routinely capturing and digesting red algae and could have commenced a longer and more sustained relationship with its prey to embark on the acquisition of photosynthesis through a secondary endosymbiosis. But what does *Goniomonas* tell us about the other, non-cryptomonad eukaryotes that also have reduced red algal endosymbionts for plastids?

Major eukaryote groups including the heterokonts/stramenopiles (algae like brown kelps, diatoms, and golden flagellates), haptophytes (abundant limestone-armoured phytoplankton whose dead ancestors comprise most of the white cliffs of Dover), dinoflagellates (including the symbionts of corals crucial for reef building and the toxic basis of certain red tides), and alveolates (parasites of animals and protists that cause diseases such as malaria and toxoplasmosis and possess relic, non-photosynthetic plastids) all harbour secondary endosymbionts of red algal origin. A long-standing debate about whether all these different types of organisms gained their red algal endosymbiont in one event—perhaps akin to the one discussed here in which a *Goniomonas*-like phagotroph captured and retained a red alga—or whether each of them descends from a separate capture of a red alga by different ancestors remains wide open, despite 40 years of investigation [8–10].

The genome of *Goniomonas* doesn’t yet resolve this debate, but it gives us an extant model with which to explore what the phagotrophic partner in the extraordinary amalgam that led to at least one group of complex algae, the cryptomonads, was like. We might just have extant descendants of the two partners—predator and prey—with which to better understand how a great swathe of eukaryotic diversity originated through secondary endosymbiosis. Maybe we should feed our *Goniomonas* on red algae rather than bacteria.
